# Spontaneous Iliopsoas Hematoma in a Patient on Acenocoumarol: Report of a Rare Case

**DOI:** 10.7759/cureus.38730

**Published:** 2023-05-08

**Authors:** Hicham El Boté, Abdelmounim Boughaleb, Jihad Lakssir, Omar Bellouki, Younes El Anbari

**Affiliations:** 1 Surgery, Regional Hospital of Beni Mellal, Beni Mellal, MAR; 2 Urology, Ibn Sina Hospital, University of Rabat, Rabat, MAR; 3 Medicine, Regional Hospital Center of Beni Mellal, Beni Mellal, MAR

**Keywords:** anticoagulant, acenocoumarol, iliopsoas hematoma, spontaneous haemorrhage, spontaneous hematoma

## Abstract

Spontaneous hematoma of the iliopsoas is a rare pathological circumstance; in the majority of cases published in the literature, it is associated with disorders of hemostasis due to anticoagulant treatment or coagulopathies. We present a case of a 64-year-old man on acenocoumarol, an anticoagulant of the vitamin K antagonist family, for atrial fibrillation, who presented with a severe left hip and flank pain with a huge ecchymosis on the left flank and a partial inability to extend the left thigh. A CT scan confirmed the diagnosis of iliopsoas hematoma. Given the hemodynamic stability of the patient, he benefited from a conservative treatment with a favourable evolution. This case highlights the underlying conditions, diagnosis, and treatment of this uncommon complication.

## Introduction

Spontaneous iliopsoas hematoma is a rare disease that is part of the pathological processes affecting this deep anatomical compartment [1]. It is often associated with hemostasis disorders related to anticoagulant therapy or coagulopathies [2].

We present the case of a 64-year-old man on acenocoumarol for atrial fibrillation who was treated for this complication. The underlying conditions, diagnosis, and treatment of this uncommon situation are briefly discussed.

## Case presentation

A 64-year-old man presented to the emergency department of our hospital for severe left hip and flank pain persisting for 5 days in the absence of a traumatic context or previous surgery. The patient had a medical history of atrial fibrillation and taking acenocoumarol 4 mg 1/2 tablet daily for 5 years.

**Figure 1:**
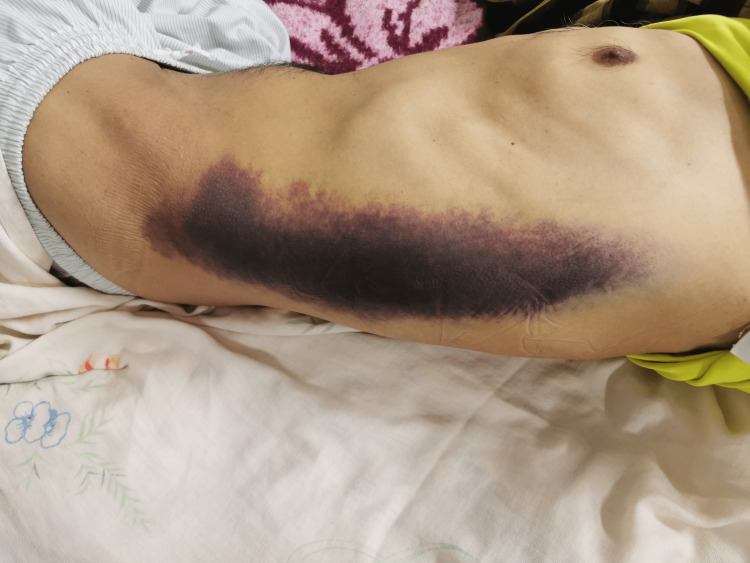
Image showing a huge ecchymosis on the left flank.

Physical examination revealed mucocutaneous pallor with a huge ecchymosis on the left flank (Figure 1) and tenderness of the left groin on palpation with a partial inability to extend the left thigh. The patient was hemodynamically stable. Intravenous paracetamol was prescribed for pain relief. Laboratory examinations revealed a hemoglobin level of 6 g/dL, a platelet count of 180× 103/mm3, and normal serum creatinine, and on check-up of hemostasis, the international normalized ratio (INR) value was 2.3.

**Figure 2:**
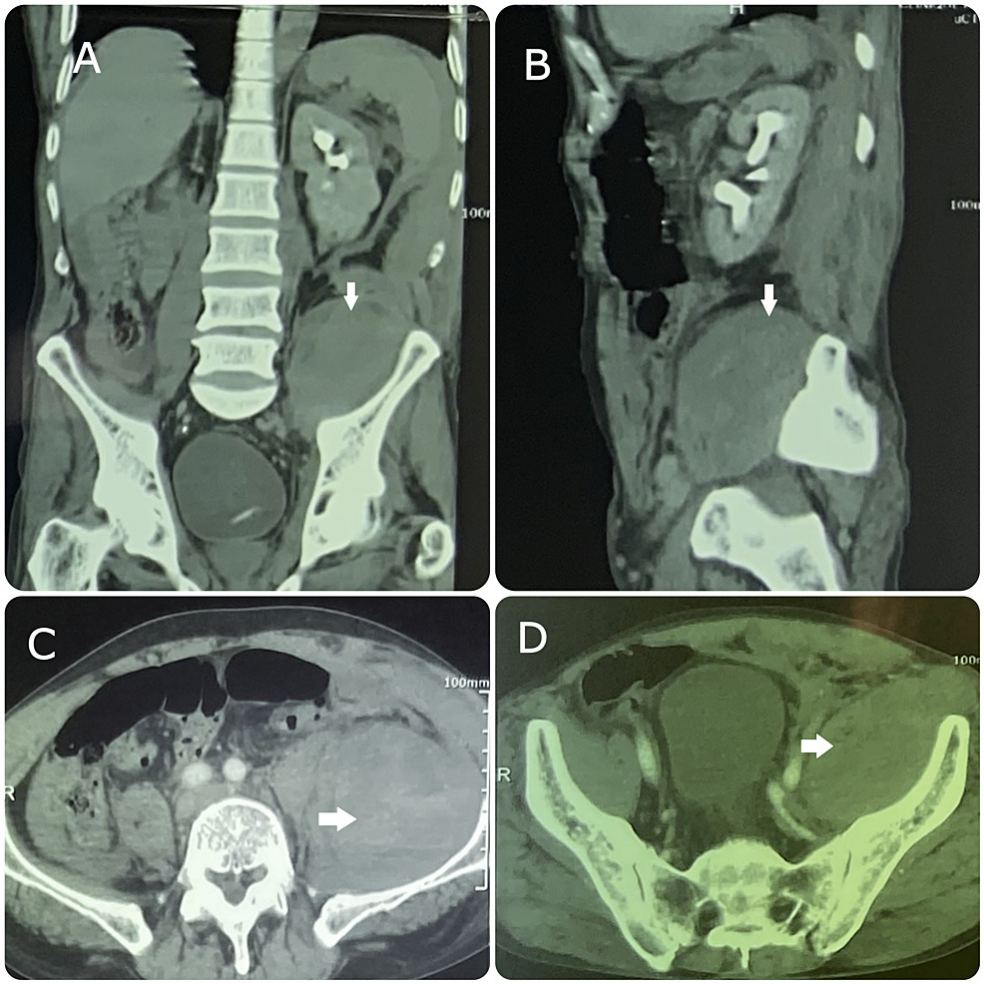
A. Coronal, B. Sagittal, and C and D. Axial abdominal computed tomography showing a large left iliopsoas muscle haematoma (arrows).

An abdominal CT scan revealed a left iliopsoas hematoma measuring approximately 9 × 10 × 18 cm (Figure 2).

In addition to the acenocoumarol suspension, the patient was transfused with 5 units of red blood cells and was closely monitored.

The CT scan was repeated one week later and no expansion of the hematoma was observed; anticoagulation was restarted.

**Figure 3:**
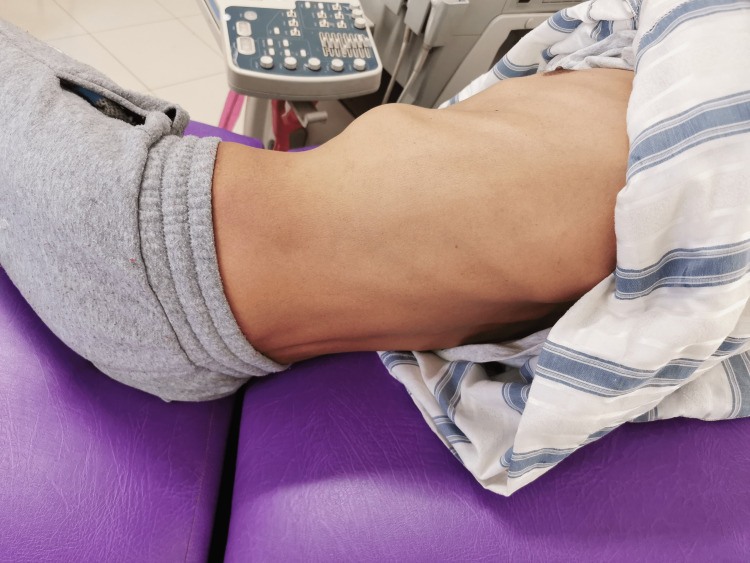
Image showing complete resorption of the ecchymosis at 1-month follow-up.

Clinical follow-up at 1 month showed complete resorption of the ecchymosis along with the resumption of normal physical activity (Figure 3).

## Discussion

The iliopsoas muscle, enveloped by its fascia, is an extraperitoneal compartment that contains the psoas major, psoas minor, and medial iliacus, which are the major flexors of the thigh and trunk [1]. Spontaneous hemorrhage in this deep anatomic entity is unusual and is defined as intramuscular bleeding without any notion of underlying trauma or prior surgery [2]. It begins at the microvascular level and increasingly affects the surrounding vessels, resulting in hematoma formation [3].

Spontaneous iliopsoas hematoma (SIPH) usually occurs in the context of anticoagulation, coagulopathies, and hemodialysis [4]. Several cases of this disorder have been reported in association with the prescription of anticoagulants during specific situations such as endovascular and cardiac procedures [5], treatment of some patients with COVID-19 [6], or in intensive care and intensive care unit patients [7]. Warfarin and heparin are the most pharmacological agents commonly implicated in these settings [8]. Haemophilia, alcoholic liver cirrhosis, chronic myeloid leukemia, and hypertensive emergencies have also been published as contributing conditions [9].

Acenocoumarol, reported in our case, is an anticoagulant of the vitamin K antagonist (VKA) family and is extensively administered in the treatment and prophylaxis of a variety of diseases including atrial fibrillation, pulmonary embolism, and valvular heart disease. It is still widely used because of its low cost in many healthcare systems. The incidence of bleeding events associated with it has been estimated in a report series to be 5.1%, dominated by gastrointestinal and cerebral haemorrhage [10]. The international normalized ratio (INR) is recommended for monitoring and dose adjustment, and the risk of major bleeding increases with increasing INR value. With an INR≥5.0, the risk of bleeding is elevated 3.6-fold compared with an INR≤2 [11]. Our patient did not have a VKA overdose and the INR value was 2.3.

The clinical manifestations of SIPH are nonspecific, such as ecchymosis or flank and possibly abdominal pain that radiates to the inguinal region. Neurological signs secondary to compression of the femoral nerve by the hematoma are of variable intensity, such as paresthesia or weakness of the extension of the thigh and leg [12]. Imaging examinations based on ultrasound, CT scan, or magnetic resonance imaging confirm the diagnosis and allow an evaluation of the volume of the hematoma [13].

Therapeutic management depends on the time of diagnosis and the haemodynamic status. If the diagnosis is early and the patient is stable, as in our case, treatment can be conservative, with the suspension of the anticoagulant, administration of an antidote in case of overdose, transfusion therapy, and appropriate analgesic treatment [14]. Embolization techniques may also be useful in the presence of active bleeding. Surgical evacuation of the haematoma is indicated in extreme cases of uncontrollable haemodynamic collapse or major neuropathic symptoms secondary to femoral nerve compression [15].

Persistent hemorrhage is an independent risk factor for short-term mortality requiring monitoring of hemoglobin levels and repeat CT scans in follow-up [16].

## Conclusions

Acenocoumarol is a conventional anticoagulant that is still widely prescribed in many countries but has a high risk of serious bleeding complications, mainly in the case of overdose.

Spontaneous iliopsoas haematoma is an exceptional complication of its administration, if undetected, it can lead to disabling neurological sequelae with permanent lower limb extension deficit, or even be life-threatening in the presence of haemodynamic instability and persistent active bleeding.
